# Pulmonary Vein Isolation with Pulsed Field Ablation and Size-Adjustable Cryo-Balloon: A Comparative Procedural Analysis of First-Time Use

**DOI:** 10.3390/jcm13113113

**Published:** 2024-05-26

**Authors:** Lyuboslav Katov, Yannick Teumer, Carlo Bothner, Wolfgang Rottbauer, Karolina Weinmann-Emhardt

**Affiliations:** Department of Cardiology, Ulm University Heart Center, Albert-Einstein-Allee 23, 89081 Ulm, Germanyyannick.teumer@uniklinik-ulm.de (Y.T.);

**Keywords:** atrial fibrillation, pulmonary vein isolation, pulsed field ablation, size-adjustable cryo-balloon

## Abstract

**Background:** Pulmonary vein isolation (PVI) is the standard of care for the treatment of symptomatic atrial fibrillation (AF). Novel techniques for PVI are the thermal size-adjustable cryo-balloon (CB) system and non-thermal pulsed field ablation (PFA) system. There are currently no data available for a direct comparison between these two systems. Furthermore, with new techniques, it is important to ensure a high level of efficiency and safety during treatment right from initial use. Therefore, the aim of this study was to directly compare the procedural data and safety of these two new PVI techniques in first-time users. **Methods:** We conducted a single-center prospective study involving 100 consecutive patients with symptomatic atrial fibrillation who underwent first-time PVI using either size-adjustable CB PVI or PFA PVI from July 2023 to March 2024. **Results:** Acute PVI was achieved in 100% of patients in both groups. First-pass isolation (FPI) was more frequently achieved in the PFA group compared to the size-adjustable CB group. The mean procedural duration and fluoroscopy dose were significantly shorter in the PFA cohort (*p* < 0.001). Furthermore, a significant reduction in fluoroscopy time was observed during the learning curve within the PFA group (*p* = 0.023). There were no major complications in both groups. **Conclusions:** Both systems demonstrate good effectiveness and safety during PVI performed by first-time users. However, the PFA group exhibited a significantly shorter procedural duration.

## 1. Introduction

The past two decades have witnessed a significant increase in scientific efforts to improve ablation technology and safety, along with a deeper investigation into the causes of arrhythmia and the development of new treatment strategies [1]. Supraventricular tachycardias impact over 1% of the population and manifest through a broad range of symptoms [2]. Among them, atrial fibrillation (AF) stands out as the most important arrhythmia encountered in clinical practice with an estimated prevalence globally reaching up to 33.5 million patients [3]. As known, AF significantly impacts both morbidity and mortality. Depending on the CHA2DS2-VASc score, anticoagulation therapy should be initiated to prevent potential complications, such as thrombus formation in the left atrium and stroke, in patients with atrial fibrillation [4,5,6]. Depending on individual related factors rhythm or rate control is aimed for AF patients [4,5,6]. Although electrical cardioversion and antiarrhythmic drug therapy are available options for rhythm control, atrial fibrillation ablation is becoming an increasingly favored method [7].

The main trigger of AF induction is widely acknowledged to be premature heartbeats originating from the pulmonary veins (PV) [8]. Therefore, pulmonary vein isolation (PVI) stands out as a pivotal strategy in rhythm management of AF patients [4,5] and it is the most frequently conducted ablation procedure in electrophysiology [7]. While 3D mapping-guided point-by-point radiofrequency (RF) ablation is the gold standard for PVI, a plethora of alternative thermal and non-thermal modalities have emerged for conducting PVI in patients with atrial fibrillation. For selected patients, however, single-shot devices can be used due to their procedural efficiency in terms of time savings for PVI. For example, the cryo-balloon (CB) as a single-shot balloon device has shown non-inferiority compared to the point-by-point RF PVI, but a significantly reduced procedure duration and thus increased procedural efficiency [9]. The latest development in the field of CB catheters is the size-adjustable CB. The balloon size of the latter catheter can be increased from 28 to 31 mm at the touch of a button. This allows the CB to be adapted during the procedure to the patient’s individual pulmonary vein anatomy [10].

Recently, pulsed field ablation (PFA), a non-thermal ablation energy, became commercially available for pulmonary vein isolation. In the ADVENT trial, Reddy et al. showed non-inferiority for PFA compared to the thermal PVI modalities [9]. It was shown that PFA PVI is associated with a shorter procedure time compared to the established, non-size-adjustable CB or point-by-point RF PVI [9].

Currently, there are no data available for a direct comparison between the latest development of the size-adjustable CB system and the PFA system for PVI. Furthermore, the evidence regarding the procedural data in first-time users of these two novel PVI modalities is sparse. For this reason, the aim of this study was to compare the procedural data and safety of these two modern techniques for PVI in first-time users.

## 2. Materials and Methods

### 2.1. Study Population

In our prospective study, we enrolled 100 consecutive patients who received PVI at Ulm University Heart Center between July 2023 and March 2024. The first 50 patients underwent size-adjustable CB PVI, whereas the next 50 patients received treatment with PFA. All operators were already well-trained in regular CB PVI technology. Blocking was applied for the two PVI technologies to minimize potential bias in the learning curve due to the non-consecutive use of a single technology. Inclusion criteria were symptomatic paroxysmal or persistent AF and a planned first-time PVI. Patients presenting with long-standing persistent AF or other left atrial arrhythmias, including those requiring procedures such as 3D mapping and RF ablation, as well as those with prior left atrial ablations, were excluded from this study. Patients under the age of 18 were excluded from this study. The data were collected prospectively as part of the ATRIUM registry (German Clinical Trials Register-ID: DRKS00013013). Each patient gave written informed consent. This research received approval from the local Ethics Committee of Ulm University and adheres to the principles delineated in the Declaration of Helsinki.

### 2.2. Periprocedural Management, Ablation Procedure and Postprocedural Management

All procedures were conducted under uninterrupted oral anticoagulation. No preprocedural cardiac imaging was performed before the PVI. After initiating deep sedation according to our standard protocol, which involved bolus of midazolam followed by continuous administration of propofol [11], a transesophageal echo probe (Philips CX50 ultrasound system, in combination with a Philips X7 TEE probe, Philips, Amsterdam, The Netherlands) was introduced. Firstly, atrial thrombus was ruled out by transesophageal echocardiography. At this point, if CB PVI was planned, an esophageal, multi-electrode, temperature probe (S-Cath, Circa Scientific LLC, Englewood, CO, USA) was trans nasally positioned at the level of the left atrium. In the case of PFA PVI, an esophageal temperature probe was not inserted. Access to the left atrium was achieved via the right groin, inferior vena cava, through the right atrium, and transseptal puncture (TSP). To achieve this, a dual puncture of the right femoral vein was performed under ultrasound guidance, followed by the placement of a steerable 10-polar coronary sinus catheter (Inquiry™ Steerable Diagnostic Catheter, Abbott, North Chicago, IL, USA). The coronary sinus catheter was positioned to record intracardiac electrical signals and provide supplementary visual guidance for the following TSP.

The TSP was performed using a non-steerable transseptal sheath in combination with a transseptal needle (CardiaGuide™ non-steerable transseptal sheath, Fixed Sheath, Biosense Webster, Irvine, CA, USA, and HeartSpan™ Transseptal Needle, Biosense Webster, Irvine, CA, USA) under fluoroscopic and transesophageal echocardiography guidance. After advancing the transseptal needle tip through the septum into the left atrium, a left atrial pressure curve was recorded. Under TEE control, a coronary guidewire (Balance Heavyweight™, Abbott, North Chicago, IL, USA) was passed through the inner lumen of the transseptal needle into the upper left pulmonary vein. Following this, the non-steerable sheath was advanced via the coronary guidewire. Once the non-steerable sheath reached the final, stable position, the transseptal needle, along with the dilator and the coronary guidewire, were retracted and removed from the patient [12]. Upon entering the left atrium, a bolus of heparin and fentanyl were administered. The target for the activated clotting time throughout the procedure was set between 300 and 350 s. During the PFA procedures, an additional bolus of atropine was administered to prevent vagal-induced, bradycardia-related complications due to sinus or atrioventricular impairment [13]. Selective pulmonary vein angiography was performed. From this point onward, the PVI procedure followed the precise protocols outlined below. Upon completion of the procedure, the ablation systems were retracted into the right atrium and then safely removed from the patient. Ultimately, closure of the puncture site was achieved using a figure-of-eight suture. Transthoracic echocardiography was utilized to rule out complications such as pericardial effusion. The patient’s neurological status was assessed upon emergence from sedation.

#### 2.2.1. Pulsed Field Ablation Protocol

**Figure 1:**
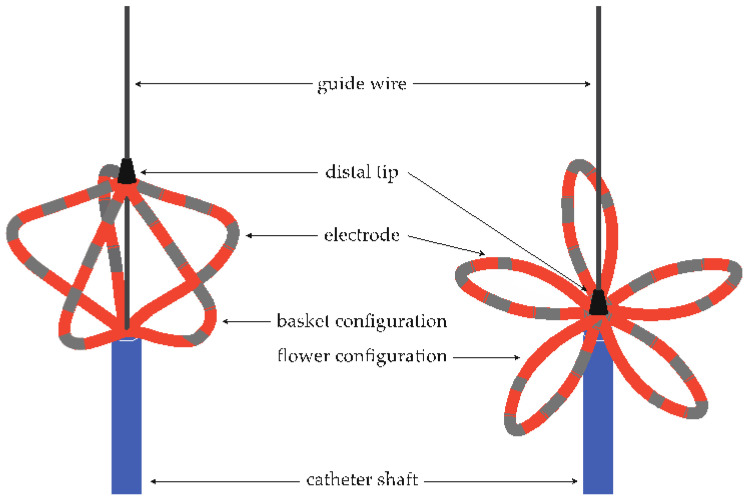
Depiction of the used PFA catheter in the basket (left side) and the flower configuration (right side).

For the PFA PVI, the non-steerable transseptal sheath was replaced by a steerable PFA sheath (Faradrive^TM^, Boston Scientific, Marlborough, MA, USA) guided by an extra-stiff guidewire (Amplatz Suport Wire Guide^TM^, Cook Group, Bloomington, IN, USA). Following this, the PFA catheter (Farawave^TM^, Boston Scientific, Marlborough, MA, USA, Figure 1 was advanced to the left upper pulmonary vein via guidewire (InQwire^TM^, Meritmedical, South Jordan, UT, USA). If necessary, an electrical cardioversion was performed to achieve sinus rhythm before ablation.

**Figure 2:**
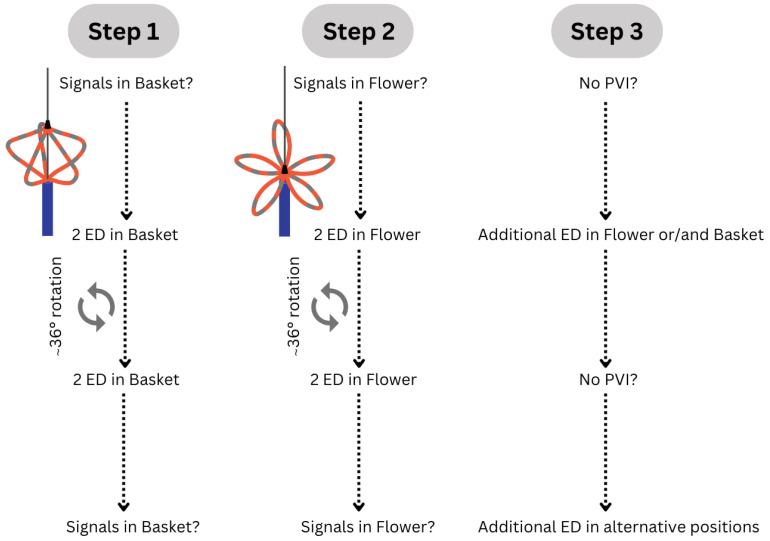
Pulsed field ablation protocol. Flowchart for the steps in the ablation with a PFA catheter. ED, energy delivery; PFA, pulsed field ablation; PVI, pulmonary vein isolation.

The PFA catheter was available in different sizes, including 31 mm and 35 mm, and features 5 splines with 4 electrodes each [14]. The energy deliveries (EDs) were routinely conducted in both the ‘basket’ and ‘flower’ configurations of the ablation catheter, according to the manufacturer’s recommendation. In both configurations, two EDs were administered in the same position, after which the ablation catheter was rotated by 36°, followed by another two EDs. In total, 8 EDs per pulmonary vein were administered, four of them in the ‘basket’ and four of them in the ‘flower’ configuration (Figure 2). The signals were documented before and after each ED. In cases where PVI was not achieved after 8 EDs, additional EDs were administered in ‘basket’, ‘flower’, or alternative positions, such as ostial EDs using, for example, an ‘olive-shaped’ configuration. The correct placement of the PFA catheter and the distance between its splines were controlled using fluoroscopy in left anterior oblique (LAO) 40° and right anterior oblique (RAO) 30° projections.

Following the full administration of all EDs in one pulmonary vein, testing for entrance and exit block was conducted. Per protocol, first-pass isolation (FPI) was defined as the immediate presence of exit and entrance block following the 8 EDs performed per pulmonary vein. After each energy delivery to the right-sided pulmonary veins, diaphragmatic movement was checked fluoroscopically to detect phrenic nerve palsy.

#### 2.2.2. Size-Adjustable Cryo-Balloon Ablation Protocol

**Figure 3:**
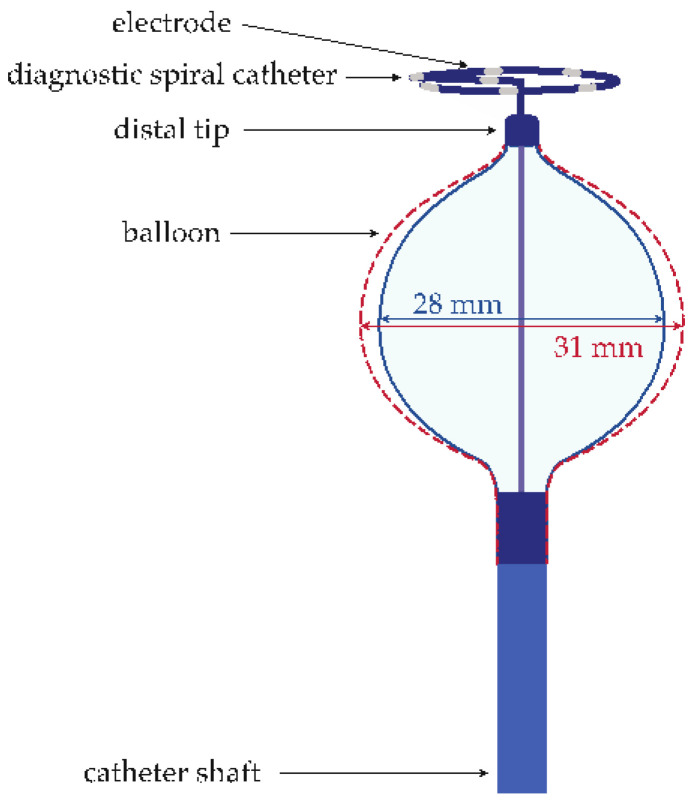
Depiction of the size-adjustable cryo-balloon.

For CB PVI, the non-steerable transseptal sheath was replaced by a steerable sheath (Polarsheath^TM^, Boston Scientific, Marlborough, MA, USA) for the size-adjustable CB (PolarX Fit™ balloon, Boston Scientific, Marlborough, MA, USA, Figure 3) via guidewire. The size-adjustable CB was advanced up to the left upper pulmonary vein. If necessary, an electrical cardioversion was performed before ablation. After balloon placement, adequate pulmonary vein occlusion and alignment were confirmed through the use of a contrast agent. For this assessment, LAO 40°, RAO 30°, and anteroposterior fluoroscopy projections were utilized. If necessary, the balloon size could be adjusted either to 28 mm or 31 mm to achieve a better occlusion and alignment. With the placement of a diagnostic octapolar, spiral catheter (Polarmap™ Circular Mapping Catheter, Boston Scientific, Marlborough, MA, USA) through the lumen of the size-adjustable CB, the pulmonary vein signals were monitored during the procedure. Subsequently, an ablation delivery was administered for 180 s. The ablation was interrupted, for example, in cases of balloon dislocation, a significant decrease in esophageal temperature below 20 °C, or in the event of phrenic nerve palsy. If the time of isolation was less than 30 s, the freeze was reduced to 120 s. If the time to isolation exceeded 60 s or could not be determined, an extra freeze was performed for 180 s. During the ablation of the right pulmonary veins, phrenic nerve stimulation was performed via the superior vena cava using the coronary sinus catheter. The detailed protocol for the ablation with the size-adjustable CB was described previously [10].

The differences between both systems (PFA system and size-adjustable CB system) are demonstrated in Table 1.

### 2.3. Statistical Analysis

Statistical analysis was conducted using SPSS^®^ Statistics (version 29.0.1.0, IBM, Armonk, NY, USA). For the categorial variables, the Chi-square test and Fisher’s exact test were utilized, and the results were presented as absolute and relative proportions. The Mann–Whitney-U test was conducted for the continuous variables. The variables were described using the median and the interquartile range (IQR). Statistical significance was determined as a *p*-value < 0.05.

## 3. Results

### 3.1. Patient Characteristics

In total, 100 patients were enrolled. Then, 50 consecutive patients (50.0%) were treated with the size-adjustable CB, and another 50 consecutive patients (50.0%) with PFA. The median (IQR) age of all patients was 71 (64; 78) years and 46 patients (46.0%) were female. They had a median CHA2DS2-VASc Score of 4 (2; 5). The median (IQR) left ventricular ejection fraction ranged from normal to mild reduced (59 (48.0; 65.0)%). More detailed baseline characteristics are summarized in Table 2.

### 3.2. Procedural Characteristics

Acute PVI was achieved in all cases within both groups. In the PFA group, FPI was attained in all pulmonary veins except for the left superior pulmonary vein (LSPV), where it was achieved in 44 patients (95.6%). In the size-adjustable CB group, the FPI rate was between 60 and 100% depending on the pulmonary vein treated. In the PFA cohort, four left common pulmonary veins (LCPV) (8.0%) were observed. In the size-adjustable CB group, there were five LCPV (10.0%) and one RMPV (2.0%). The large balloon configuration was used in 8 out of 45 pulmonary veins at the LSPV, 7 out of 45 at the LIPV, 11 out of 50 at the RIPV, 8 out of 50 at the RSPV, and all five pulmonary veins at the LCPV, but not at the RMPV. Detailed information is provided in Table 3.

**Figure 4:**
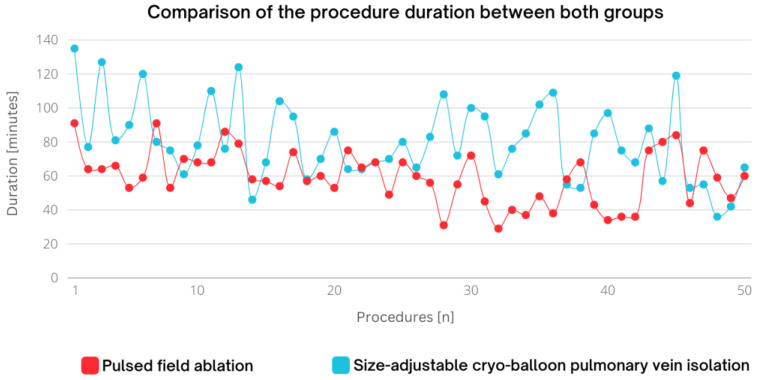
Comparison of the procedure duration between both groups (*p* < 0.001). In red, all pulsed field ablation procedures are depicted, and in blue, all size-adjustable CB procedures are depicted. CB, cryo-balloon.

The median (IQR) procedure duration for the patients treated with PFA was 59 (47.7; 68.5) min. The median (IQR) fluoroscopy time and dose were 18.5 (16.0; 23.2) min and 1170.4 (831.2; 1852.6) cGy·cm^2^, respectively. The median (IQR) dwell time in the PFA group was 32.5 (25.0; 44.2) min. In the CB cohort, the mean (IQR) values for procedural duration (76.5 (64; 95.5) min) and fluoroscopy dose (2364.8 (1376.8; 4566.9) cGy·cm^2^) were significantly higher (*p* < 0.001) (Figure 4, Table 4). The mean (IQR) fluoroscopy time among the patients treated with the size-adjustable CB system was lower (17.7 (12.5; 22.0) min; *p* = 0.188).

A disparity was noted between the two groups in terms of the volume of contrast agent administered during the procedure (*p* = 0.003). The median (IQR) contrast agent volume was higher in the patients treated with the size-adjustable CB (90.0 (30.0; 140.0) ml). Detailed procedural data are provided in Table 4.

For a more detailed evaluation of the learning curve, we examined the first and last five procedures carried out by three distinct first-time users, all of whom were previously unfamiliar with the systems. This analysis unveiled a notable reduction in fluoroscopy time within the PFA group (Table 5).

In the size-adjustable CB cohort, no significant differences were observed between the initial and final procedures, except for contrast agent volume (*p* = 0.013). The remaining procedural data, including a presentation of the learning curve in that group, are presented in Table 6.

**Figure 5:**
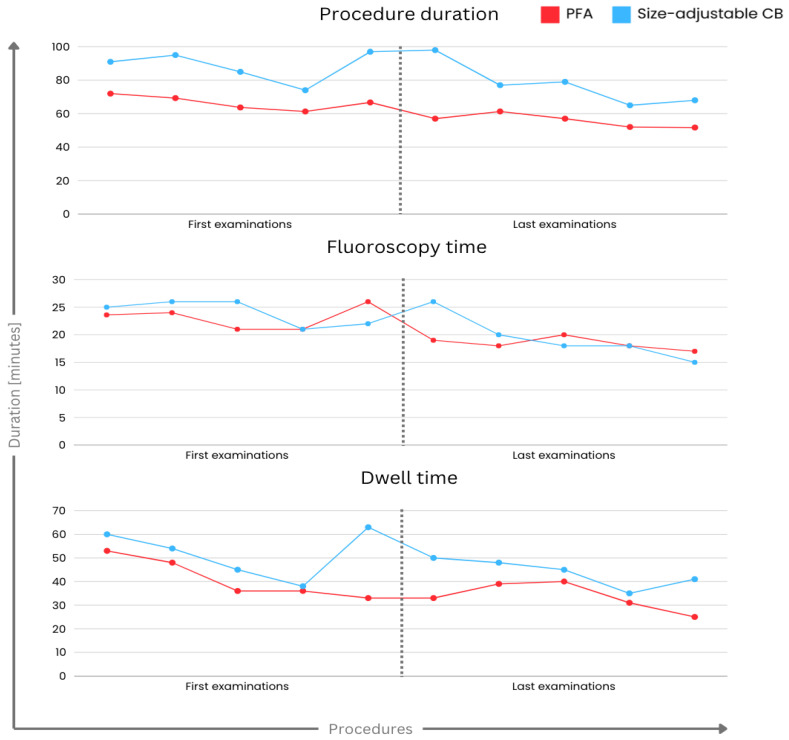
Depiction of the learning curve during PFA PVI and size-adjustable CB PVI regarding procedure duration, fluoroscopy time and dwell time. On the left side, presentation of the average values of the first five examinations conducted by three examiners with the PFA (red) or the size-adjustable CB (blue) system. On the right side, presentation of the average values of the last five examinations. CB, cryo-balloon; PFA, pulsed field ablation; PVI, pulmonary vein isolation.

The learning curves for procedural duration, fluoroscopy and dwell time, conducted by first-time examiners, using PFA and size-adjustable CB, are presented in Figure 5.

### 3.3. Safety

Regarding procedural safety, no vascular access complications, air embolism, pericardial effusion/tamponade, persistent phrenic nerve palsy, or strokes/transient ischemic attacks were detected. In two patients (4%), freezing at the right pulmonary veins with the size-adjustable CB was interrupted immediately due to phrenic nerve palsy. By the end of the procedure, the phrenic nerve palsy had resolved spontaneously in both patients. In one CB PVI patient (2.0%), post-interventional pericarditis was observed, with rapid improvement within 24 h after administration of a nonsteroidal anti-inflammatory drug. During the PFA procedures, no complication was observed. There were no fatalities in both groups. In summary, no statistical significance was observed regarding all complications (*p* = 0.213). Detailed information is provided in Table 7.

## 4. Discussion

As far as our knowledge extends, there is no existing evidence directly comparing the latest advancements in size-adjustable CB PVI and PFA PVI. Specifically, there is a notable dearth of evidence regarding procedural data and safety comparisons during the initial implementation of these two single-shot PVI modalities. Hence, the objective of this study was to conduct a direct comparison of procedural data and assess the safety profiles of these devices in first-time users.

### 4.1. Efficiency

When considering the efficiency of a PVI procedure, the duration from skin-to-skin plays a central role in daily routine. PFA PVI demonstrated faster PVI and shorter procedure duration compared to non-size-adjustable CB systems [9,15]. As our study shows, this result appears to be transferable to size-adjustable CB systems in comparison to PFA PVI.

Looking at the procedure duration of first-time users in our study, it is intriguing to observe that the duration of the procedure aligns numerically with the data presented in the large MANIFEST-PF registry and the FARA-Freedom study concerning PFA PVI [16,17,18]. Additionally, in our study, the initial adoption of the size-adjustable CB system resulted in a shorter procedure duration, in contrast to the findings from the FROZEN-AF study, where only a minority of patients were treated using this novel system [19]. Similarly, the procedural duration is consistent, or slightly longer, with other non-size-adjustable CB studies [20]. From our perspective, experienced non-size-adjustable CB users demonstrate proficiency in efficiently performing PVI with both novel systems right from their initial uses. Regarding the learning curve within the system, PFA technology appears to provide more consistent procedure durations across all patients, reaching a plateau at about two-thirds of the learning curve. In contrast, the procedure duration for the CB system seems more variable throughout the cases, exhibiting a less steady learning curve. We attribute this observation to the relatively long energy delivery times required by the CB technology compared to PFA technology. Missed first-pass isolations result in extra energy deliveries, significantly extending the procedure duration in CB PVI.

The disparity in procedure duration between PFA technology for PVI and the size-adjustable CB is coupled with a significantly shorter dwell time of the ablation catheter within the left atrium. This difference can be attributed to the fact that PFA technology requires mere seconds for ED to achieve PVI, contrasting with the minutes per freeze needed for size-adjustable CB PVI. Even with a TTI-based and optimized ablation protocol for the size-adjustable CB employed in our study [10], a significant gap in procedure duration between CB and PFA PVI persists. Additionally, achieving optimal pulmonary vein occlusion is crucial with the size-adjustable CB to attain PVI, necessitating verification through contrast agent injection at each occlusion attempt. In contrast, for PVI with the PFA technique, tissue contact at the pulmonary vein ostium is vital for optimal transmural lesion formation [21]. Tissue contact with the PFA system is confirmed via tactile and visual feedback, eliminating the need for pulmonary vein occlusion verification through contrast agent injection as an additional procedural step. We believe these factors contribute to the shorter procedure duration and reduced left atrium dwell time associated with PFA technology.

Another benefit of eliminating the necessity for pulmonary vein occlusion verification through contrast agent injection in PFA PVI is the reduction in the volume of contrast agent needed for the procedure. The total volume of contrast agent used in this study is relatively high [22,23]. This finding is attributed to a pulmonary vein angiography prior ablation with both systems at our center. The lower consumption of contrast agent in the PFA group, compared to the size-adjustable CB group, can be explained by the additional mandatory procedural step of verifying sufficient pulmonary vein occlusion through contrast agent injection in CB PVI. However, during the learning curve with the size-adjustable CB, there was a significant reduction in contrast agent usage when comparing the initial to the final evaluated examinations. Consequently, the high amount of contrast agent is partly attributed to a learning curve effect, which was mitigated as examiners became more proficient with the size-adjustable CB system. In contrast, there was no learning curve regarding contrast agent usage with the PFA system. In PFA PVI, a contrast agent is only used for pulmonary vein angiography.

Both single-shot devices are fluoroscopy-guided. In our study, the mean fluoroscopy time did not show a significant difference between both groups. However, the fluoroscopy dose was notably higher in the size-adjustable CB group. When assessing pulmonary vein occlusion with the CB, it is recommended to use high-resolution fluoroscopic imaging to accurately depict a potential contrast agent outflow, which typically leads to a higher fluoroscopy dose. However, the PFA PVI does not solely offer advantages in its current state of development. The PFA system also exhibits a comparatively high fluoroscopy time and dose compared to RF-based PVI guided by 3D mapping [9]. PFA catheter positioning relies heavily on fluoroscopy. Each position of the PFA system must be assessed in two orthogonal fluoroscopic views and re-evaluated after every energy delivery. Moreover, to ensure adequate PVI, the PFA catheter needs to be repositioned multiple times under fluoroscopic guidance between each energy delivery to isolate the entire pulmonary vein circumference. This challenge could potentially be addressed in the future by integrating the PFA technique into 3D mapping technology.

### 4.2. Safety

In theory, due to the non-thermal ablation energy and different threshold for irreversible electroporation depending on the tissue, the risk profile of PFA PVI is different from that of thermal PVI modalities, like the CB PVI [24]. This can be the reason for the numerical higher phrenic nerve palsy rate in the size-adjustable CB group in this study compared to the PFA group. This observation is also consistent with registry studies on CB PVI and PFA PVI [25,26]. Although it is necessary to stop energy delivery in case of impending phrenic nerve injury during CB PVI, sufficient PVI is still achievable [27]. Thus, there appears to be no elevated incidence of phrenic nerve palsy even during the initial utilization of these PVI modalities. However, it is worth noting that transient phrenic nerve palsy associated with PFA has also been documented [16].

While no pericarditis was detected in the PFA group, symptomatic pericarditis was detected in the CB group in our study. Based on current data, this observation does not seem coincidental but rather a consequence of the ablation energy employed [16,28,29].

As previously outlined by Badertscher et al., comparing non-adjustable CB and PFA, the current study also revealed no vascular access complications within both groups [14]. Moreover, the potential benefits of utilizing a single venous puncture during PFA procedures to further minimize possible complications were demonstrated by Tilz et al. [30]. In contrast, a second venous puncture is needed in CB PVI due to safety reasons. The coronary sinus catheter is crucial for phrenic nerve stimulation during ablation of the right PVs during CB PVI. Thus, a single-groin-puncture procedure should not be performed.

Recent studies report less PV narrowing post-PVI using PFA compared to thermal ablation strategies. Due to lack of periprocedural imaging, we cannot evaluation this finding in our cohort [31].

It is worth mentioning that a PFA PVI procedure could be performed completely without using contrast agent since the position of the PFA system can be assessed solely by fluoroscopy. Contrast agent administration could be avoided during PFA PVI, particularly in instances of poor renal function or abnormal thyroid levels.

In summary, the complication rates observed in this study are in line with existing data despite the first-time use of these modalities [16,32]. Therefore, the use of the size-adjustable CB and PFA system can be considered safe in first-time use.

## 5. Conclusions

In conclusion, our study highlights the comparability of size-adjustable CB PVI and PFA PVI in procedural efficiency and safety beginning from the first procedure performed. Nonetheless, PFA PVI presents several advantages, including reduced procedural duration, minimized contrast agent usage, and the potential for fewer complications.

## Figures and Tables

**Table 1:** Comparison of the differences between the two systems.

System Characteristics	Pulsed Field Ablation	Size-Adjustable Cryo-Balloon
Mechanism of ablation	Selective cell electroporation	Unselective freeze
Energy form	Non-thermal	Thermal
Device diameter	31 mm or 35 mm ^1^	28 mm–31 mm ^2^
Device configuration during energy delivery	‘basket’ and ‘flower’, as well as every position in between (if necessary)	Oval
Energy delivery	At least 8 applications per vein with duration 2.5 s each	At least 1 application per vein with duration 180 s (if TTI < 30 s 120 s)
Signals monitoring in the pulmonary veins	20 electrodes	8 electrodes

^1^ size of the catheter must be selected before the procedure; ^2^ balloon size can be changed during the procedure.

**Table 2:** Baseline characteristics of the patients treated with pulsed field ablation and the size-adjustable cryo-balloon.

Baseline Characteristics	All Patients (*n* = 100)	Pulsed Field Ablation (*n* = 50)	Size-Adjustable Cryo-Balloon (*n* = 50)	*p*-Value
Age [years], median (IQR)	71 (64; 78)	70 (62; 77)	72 (65; 79)	0.546
Female, *n* (%)	46 (46.0)	22 (44.0)	24 (48.0)	0.688
BMI [kg/m^2^], median (IQR)	27.3 (24.2; 30.4)	27.6 (27.6; 30.4)	26.7 (23.9; 30.8)	0.804
CHA_2_DS_2_-VASc score, median (IQR)	4 (2; 5)	4 (2; 5)	4 (2; 4)	0.283
LA diameter [mm], median (IQR)	45 (40; 50)	45 (40; 50)	45 (42; 50)	0.095
LAVI [mL/m^2^], median (IQR)	39.8 (29.8; 49.4)	39.4 (29.7; 49.9)	37.0 (27.6; 48.2)	0.382
LVEF (%), median (IQR)	59 (48.0; 65.0)	59 (48.0; 65.0)	59 (46.0; 65.0)	0.670
Hypertension, *n* (%)	77 (77.0)	40 (80.0)	37 (74.0)	0.476
Diabetes mellitus, *n* (%)	16 (16.0)	11 (22.0)	5 (10.0)	0.102
Hyperlipoproteinemia, *n* (%)	79 (79.0)	38 (76.0)	41 (82.0)	0.461
Coronary artery disease, *n* (%)	49 (49.0)	24 (48.0)	25 (50.0)	0.841
OSA, *n* (%)	6 (6.0)	2 (4.0)	4 (8.0)	0.400
Prior Stroke/ TIA, *n* (%)	10 (10.0)	4 (8.0)	6 (12.0)	0.452

BMI, body mass index; IQR, interquartile range; LA, left atrial; LAVI, left atrial volume index; LVEF, left ventricular ejection fracture; OSA, obstructive sleep apnea.

**Table 3:** Ablation parameters of the patients treated with pulsed field ablation and the size-adjustable cryo-balloon.

Ablation Parameters	Pulsed Field Ablation (*n* = 50)	Size-Adjustable Cryo-Balloon (*n* = 50)	*p*-Value
Acute PVI, *n* (%)	50 (100)	50 (100)	1.000
FPI LSPV, *n* (%)	44 (95.6)	41 (91.1)	0.434
FPI LIPV, *n* (%)	46 (100)	40 (88.9)	0.026
FPI RIPV, *n* (%)	50 (100)	43 (86.0)	0.012
FPI RSPV, *n* (%)	50 (100)	43 (86.0)	0.012
FPI LCPV, *n* (%)	4 (100)	3 (60.0)	0.444
FPI RMPV, *n* (%)	N/A	1 (100)	N/A
ED per patient, median (IQR)	32.0 (32.0; 32.0)	6.0 (6.0; 8.0)	N/A

ED, energy deliveries, FPI, first-pass isolation; IQR, interquartile range; LCPV, left common pulmonary vein; LIPV, left inferior pulmonary vein; LSPV, left superior pulmonary vein; N/A, not applicable; PVI, pulmonary vein isolation; RIPV, right inferior pulmonary vein; RMPV, right medial pulmonary vein; RSPV, right superior pulmonary vein.

**Table 4:** Procedure characteristics of the patients treated with pulsed field ablation and the size-adjustable cryo-balloon.

Procedure Characteristics	Pulsed Field Ablation (*n* = 50)	Size-Adjustable Cryo-Balloon (*n* = 50)	*p*-Value
Procedure duration [min], median (IQR)	59.0 (47.7; 68.5)	76.5 (64.0; 95.5)	< 0.001
Dwell time [min], median (IQR)	32.5 (25.0; 44.2)	41.0 (32.0; 55.0)	0.002
Fluoroscopy time [min], median (IQR)	18.5 (16.0; 23.2)	17.7 (12.5; 22.0)	0.188
Fluoroscopy dose [cGy·cm^2^], median (IQR)	1170.4 (831.2; 1852.6)	2364.8 (1376.8; 4566.9)	< 0.001
Contrast agent [mL], median (IQR)	30.0 (30.0; 90.0)	90.0 (30.0; 140.0)	0.003

IQR, interquartile range.

**Table 5:** Procedure characteristics of the patients treated with pulsed field ablation comparing the first and last procedures conducted by first-time examiners.

PFA Procedure Characteristics	First Examinations	Last Examinations	*p*-Value
Procedure duration [min], median (IQR)	64.0 (57.0; 74.0)	58.0 (38.0; 75.0)	0.112
Dwell time [min], median (IQR)	40.0 (32.0; 50.0)	34.0 (22.0; 45.0)	0.268
Fluoroscopy time [min], median (IQR)	22.3 (18.6; 27.0)	18.0 (13.1; 23.6)	0.023
Fluoroscopy dose [cGy*cm^2^], median (IQR)	1630.0 (973.0; 2062.0)	882.0 (611.0; 1144.4)	0.085
Contrast agent [mL], median (IQR)	30.0 (30.0; 90.0)	30.0 (30.0; 42.5)	0.906

IQR, interquartile range.

**Table 6:** Procedure characteristics of the patients treated with size-adjustable cryo-balloon comparing the first and last procedures conducted by first-time examiners.

Size-Adjustable Cryo-Balloon Procedure Characteristics	First Examinations	Last Examinations	*p*-Value
Procedure duration [mins], median (IQR)	80.0 (75.0; 110.0)	80.0 (55.0; 95.0)	0.345
Dwell time [mins], median (IQR)	45.5 (38.0; 63.7)	41.0 (28.0; 56.0)	0.505
Fluoroscopy time [min], median (IQR)	21.1 (19.5; 29.8)	17.8 (12.7; 22.1)	0.137
Fluoroscopy dose [cGy*cm^2^], median (IQR)	2577.6 (1869.0; 6570.6)	1735.2 (1301.0; 4613.0)	0.367
Contrast agent [mL], median (IQR)	140.0 (90.0; 140.0)	40.0 (35.0; 90.0)	0.013

IQR, interquartile range.

**Table 7:** Complications in the patients treated with pulsed field ablation and the size-adjustable cryo-balloon.

Complications	Pulsed Field Ablation	Size-Adjustable Cryo-Balloon	*p*-Value
Transient phrenic nerve palsy, n (%)	0 (0)	2 (4.0)	
Persistent phrenic nerve palsy, n (%)	0 (0)	0 (0)	
Pericarditis, n (%)	0 (0)	1 (2.0)	
Pericardial effusion/ tamponade, n (%)	0 (0)	0 (0)	0.213
Vascular access complications, n (%)	0 (0)	0 (0)	
Air embolism, n (%)	0 (0)	0 (0)	
Strokes/transient ischemic attacks, n (%)	0 (0)	0 (0)	

## Data Availability

The data presented in this study are available on request from the corresponding author. The data are not publicly available due to data privacy laws.
